# Delayed Metastasis of Clear Cell Renal Carcinoma to the Colon in the Setting of Benign Kidney Disease

**DOI:** 10.7759/cureus.22659

**Published:** 2022-02-27

**Authors:** Ariana R Tagliaferri, Caitlyn Costanzo

**Affiliations:** 1 Internal Medicine, St. Joseph’s Regional Medical Center, Paterson, USA; 2 Colon and Rectal Surgery, Sidney Kimmel Medical Center Thomas Jefferson University, Philadelphia, USA; 3 Colorectal Surgery, Thomas Jefferson University Hospital, Philadelphia, USA

**Keywords:** polycystic kidney disease, kidney transplantation, colorectal surgery, colonic neoplasms, renal cell carcinoma

## Abstract

Clear cell renal carcinoma (CCRC) is a common variant of renal cell carcinoma (RCC), which presents with unpredictable features. The occurrence of RCC in those with autosomal dominant polycystic kidney disease (ADPKD) is debated. Most studies agree that ADPKD does not increase the risk of RCC; however, it makes diagnosing RCC difficult due to the nature of the disease. RCC frequently metastasizes to the lungs, lymph nodes, bones, liver, adrenal glands, and brain, but rarely metastasizes to the colon. In all previous reports, primary RCC was already diagnosed in the kidneys; thus, metastatic CCRC to the colon has never been described in the current literature in the absence of a primary renal tumor.

Here, we report a rare presentation of metastatic CCRC wherein a patient with ADPKD presented with an obstructing sigmoid mass six years after bilateral nephrectomy for pathologically benign cysts. Despite a close follow-up after nephrectomy, our patient’s non-specific symptoms were attributed to underlying comorbidities and more likely etiologies of back pain, diarrhea, and anemia, thus delaying and complicating the diagnosis of CCRC which subsequently led to metastases at the time of presentation.

Although past literature has described CCRC metastases to other parts of the gastrointestinal tract or even described primary clear cell carcinoma of the colon, this is the first case in which a patient with benign cystic renal disease developed CCRC presenting as metastatic disease of the colon, rectum, liver, and lung. This paper will address the manifestations of ADPKD and postulate mechanisms for the unpredictable nature of this patient’s RCC metastasis.

## Introduction

Autosomal dominant polycystic kidney disease (ADPKD) is an inherited disease characterized by bilateral renal and liver cysts, resulting in end-stage renal failure [1]. In the early stages, it can be difficult to distinguish between renal cell carcinoma (RCC) and ADPKD clinically. This is because end-stage renal disease (ESRD) is the most common manifestation of ADPKD, which often mimics a premalignant condition giving rise to widespread systemic disease [1,2]. RCC rarely presents with the classical “hematuria, flank pain and palpable mass,” but rather as non-specific systemic symptoms [1]. When RCC occurs in ADPKD patients, it typically presents at a young age and more commonly as bilateral tumors of the sarcomatous or multicentric subtype [2-4].

Due to the high prevalence in males, the development of RCC is thought to be related to changes in growth factors and androgen excess in end-stage kidneys [5]. Although the hallmark feature of ADPKD is the marked proliferation of expanding cysts, RCC does not have an increased prevalence in ADPKD patients compared to the general population [1]. Because the prevalence of the ADPKD gene is 1:1,000 and the age-adjusted incidence of RCC is 5-6/100,000 per year in the general population, the possibility of concomitant occurrence of both conditions is not inconceivable but uncommon [1,3]. Some feel that patients with ADPKD are at higher risk due to the development of ESRD, requiring transplantation and/or dialysis, as previous studies have shown RCC to present many years after transplantation or dialysis in the general population [2]. On the contrary, many simply recognize the radiological and clinical challenges in diagnosing RCC in the setting of ADPKD and feel that there is only an apparent increased incidence due to more frequent radiographic surveillance [2]. The presence of complex cysts, proteinaceous debris, infection, and bleeding in ADPKD mimic the appearance of RCC on computed tomography (CT) scans and complicate the diagnosis of RCC preoperatively [2]. Recent studies have advocated for the use of renal arteriography for the diagnosis of RCC in those with ADPKD due to the high sensitivity and specificity for malignancy [1].

RCC frequently metastasizes to the lungs, lymph nodes, bones, liver, adrenal glands, and brain, but rarely to the colon [6]. In all previous reports, primary RCC was already diagnosed in the kidneys, and thus metastatic CCRC to the colon has never been described in the current literature in the absence of a primary renal tumor [6,7]. Here, we report a rare presentation of metastatic clear cell renal carcinoma (CCRC), in which a patient with ADPKD presented with an obstructing sigmoid mass six years after bilateral nephrectomy for pathologically benign cysts.

## Case presentation

History of presenting illness

**Figure 1:**
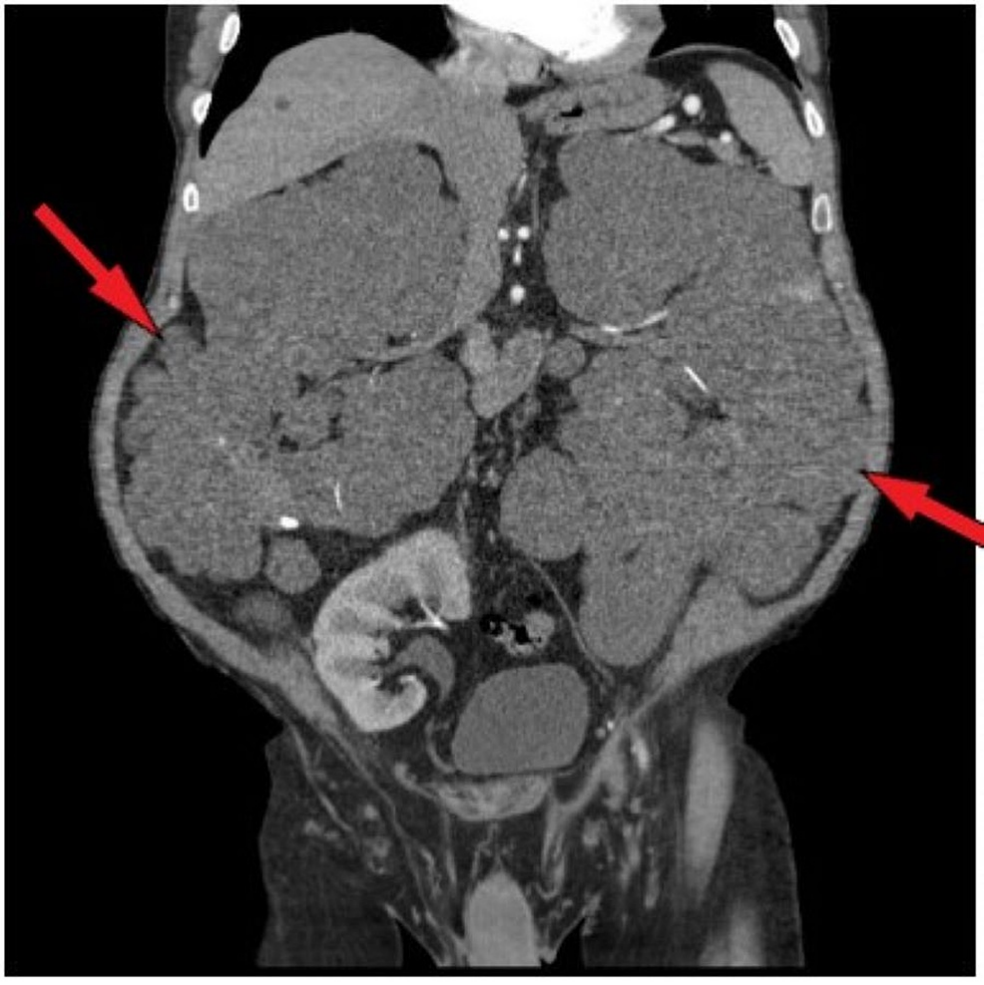
CT scan (2012), coronal view of enhancing cysts of the native kidneys. Left kidney: inner polar region enhancing mass measuring 4.5 cm (red arrow). Right kidney: inner polar region enhancing mass measuring 8.0 cm (red arrow). CT: computerized tomography

Here, we report the case of a 77-year-old man with ADPKD and ESRD who was status post-deceased donor renal transplantation and on chronic immunosuppression with mycophenolate and cyclosporine for 15 years prior to presentation. The patient was only taking prednisone at the time of presentation. Over the course of 10 years, he developed bilateral renal masses in his native kidneys and worsening renal failure (Figure 1), requiring laparoscopic bilateral nephrectomy. The final pathology determined the masses to be benign cysts.

Six years following the bilateral nephrectomy, he presented for a screening colonoscopy, during which there was a failure to pass beyond the sigmoid colon due to due to a friable and obstructing mass. Upon further questioning, the patient reported a six-month history of blood per rectum and diarrhea. The patient had three previously normal colonoscopies performed for routine screening.

Radiological and laboratory findings

**Figure 2:**
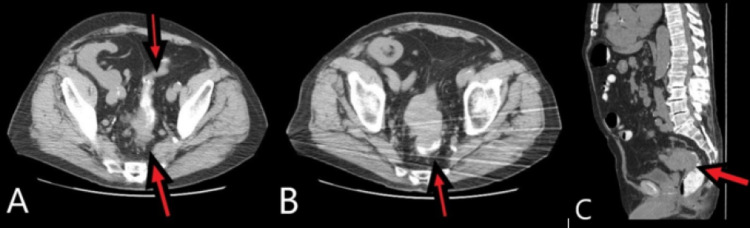
Rectosigmoid malignancy with extension into the peritoneum. (A) Preoperative CT scan, axial view of an 8.8 cm mass at the rectosigmoid junction (red arrow). (B) Preoperative CT scan, axial view of an 8.8 cm mass at the rectosigmoid junction (red arrow). (C) Preoperative CT scan, sagittal view of locoregional spread and lymphovascular invasion (red arrow). CT: computerized tomography

A follow-up CT scan illustrated an 8.8 cm mass at the rectosigmoid junction (Figure 2).

**Figure 3:**
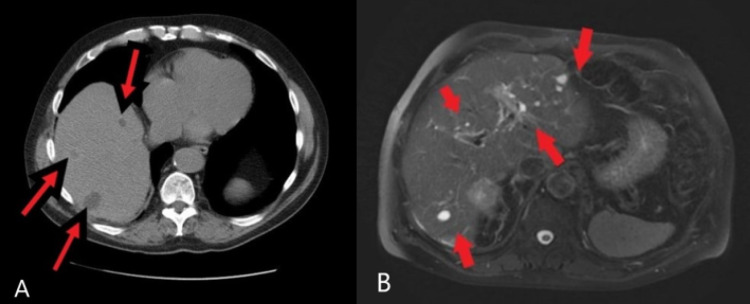
Hepatic metastases and subcapsular metastases on CT and MRI. (A) Preoperative CT scan, axial view of vague hypodensities in segments six and seven in the liver which are not dense enough to represent cysts. Concerning for metastatic disease (red arrow). (B) Preoperative MRI of the liver, innumerable cysts, multiple hypoenhancing and hyperenhancing lesions read as hepatic metastases, as well as subcapsular metastases (red arrow). CT: computerized tomography; MRI: magnetic resonance imaging

A hypodensity in segment six of the liver was concerning for metastatic disease, and subsequent magnetic resonance imaging (MRI) revealed numerous, diffuse, subcapsular metastases with variable enhancement (Figure 3). The largest lesion measured up to 2.9 × 2.9 cm.

The patient’s carcinoembryonic antigen was elevated at 6.2 ng/mL. At this point, the working diagnosis was stage IV near-obstructing, bleeding sigmoid colon cancer. According to Medical Oncology, the patient was not a candidate for chemotherapy after having undergone years of immunosuppressant therapy after transplantation. Cabozantinib was initiated; however, due to worsening kidney function, diarrhea, and hypertension, the patient was expedited for surgical treatment exclusively.

Surgical and immunohistology findings

**Figure 4:**
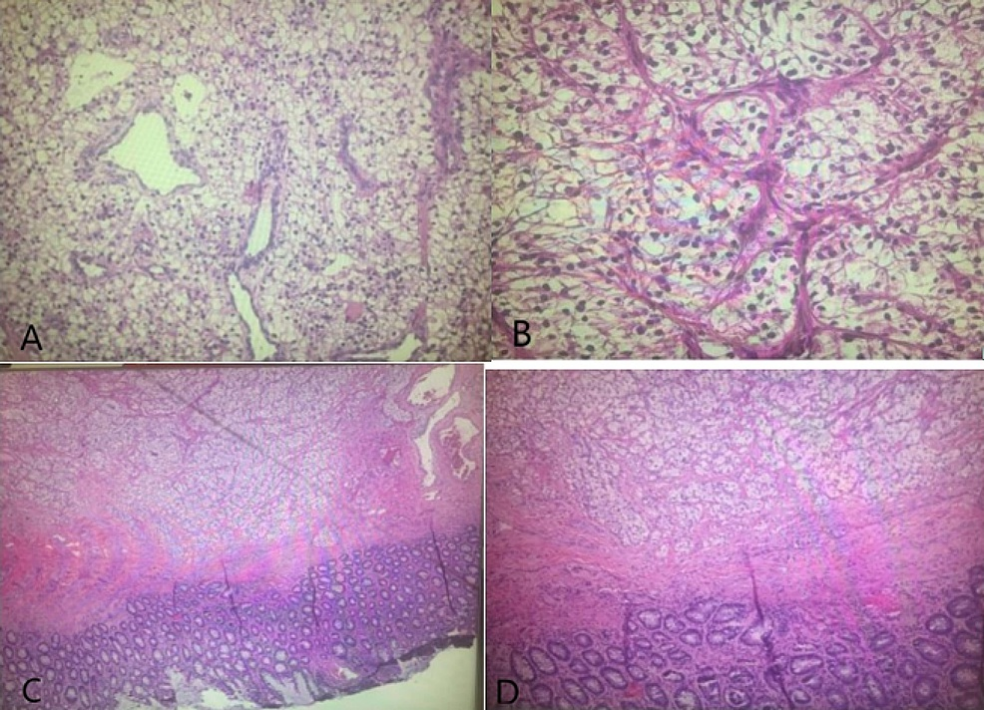
Surgical specimens from the sigmoid mass revealing clear cell renal carcinoma. (A) Rectal tumor tissue showing clear cell renal carcinoma, (B) perirectal fibroadipose tissue with multiple clear cell renal nodules, and (C and D) peritoneal tissue showing metastatic renal cell carcinoma, clear cell type.

The patient underwent an exploratory laparotomy, low anterior resection with liver, and peritoneal biopsies. The surgeons found a large mid-rectal cancer appearing to be perforated into the cul-de-sac, multiple lesions in the liver, and peritoneal cysts posterior to the right lobe of the liver. Pathology reports concluded metastatic renal cell carcinoma, clear cell type found within all biopsied tissue, which was shown to be positive for paired box gene 2 (PAX-2), cluster of differentiation 10 (CD-10), and cancer antigen 9 (CA-9) on immunohistochemical stains (Figure 4).

**Figure 5:**
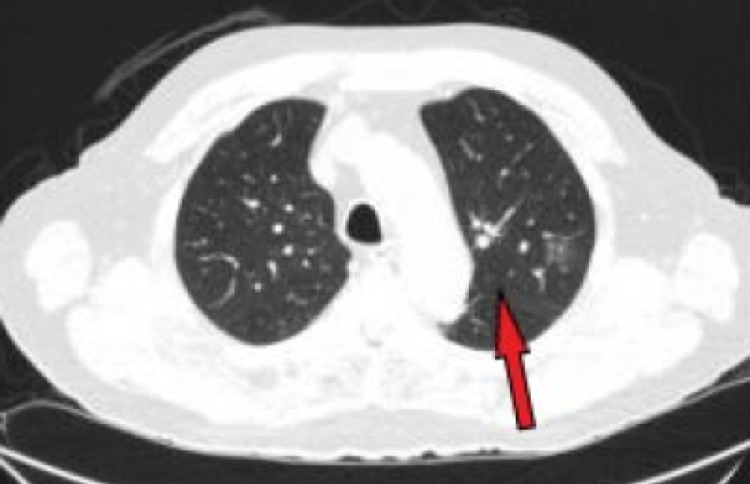
Postoperative CT demonstrating lung metastases. Non-contrast CT, axial views with two left lung hyperdensities, representing metastatic nodules (red arrow). CT: computerized tomography

Six regional lymph nodes were recovered and tested negative for tumor occurrence. Pathology from his transplanted kidneys was reviewed at this time, revealing interstitial fibrosis and tubular atrophy from cyclosporin toxicity; however, no neoplasm was identified. The patient had an uneventful postoperative recovery and was redirected to the care of Medical Oncology. Of note, the patient underwent a non-contrast CT scan as an outpatient with Oncology, where metastatic pulmonary nodules were found (Figure 5).

## Discussion

The first question was to determine if the patient had primary clear cell adenocarcinoma of the rectum, or if this was truly metastatic RCC from the native kidneys removed in 2012. Distinguishing between the two entities is vital for therapeutic direction [8]. Although both tumors contain clear cells, RCC cells mainly form nests or acini-form structures with vascular sinusoids. In contrast, cells of rectal clear cell carcinoma mostly arrange in glandular tube-like structures, similar to traditional adenomas [8]. Rectal clear cell carcinoma shows cytokeratin (CK)20 positivity and CK7 negativity. In comparison, RCC is only positive for CD10 and vimentin [9]. This was the case in our patient, whose histology and staining patterns were consistent with metastatic renal clear cell carcinoma from his native kidneys.

Up to one-third of patients with CCRC will have metastatic disease at the time of diagnosis [4,10]. Many individuals will have metastatic disease in multiple organ sites, with a mean of four organs involved; however, it is not common for RCC to metastasize to the colon or rectum [4,10]. Our patient had pulmonary, hepatic, and colorectal metastases at the time of diagnosis. The colorectal involvement manifested as obstruction and bleeding. The most common site of gastrointestinal RCC metastasis is the stomach, although the prevalence is so low that the probability of a second primary tumor is more likely than the occurrence of RCC metastasis to the stomach [9]. It is estimated that small intestine involvement is only present in 3-4% of patients with RCC [10]. Of the few cases involving the gastrointestinal tract, there have only been 10 cases of metastatic RCC presenting with lower gastrointestinal bleeding, and none of those cases could confirm the presence of metastasis as a result of direct invasion [8,11]. The exact incidence of colonic involvement in the general population is unknown [10]. Approximately 61 cases of rectal metastasis of CCRC in ADPKD patients have been recorded since 1954; however, all patients were previously diagnosed with primary tumors of the kidneys [3,9]. Our case is very rare because our patient had benign kidney disease before being diagnosed with metastatic CCRC to his colon, rectum, liver, and lungs.

The exact mechanism of this metastasis is unclear but can be explained as the result of diffuse peritoneal seeding from an intra-abdominal or retroperitoneal tumor [6]. However, the blood supply of the rectum is not compatible with the hematogenous spread of RCC [6], which is why the small intestine is more commonly affected than the colon. Although case reports have proposed direct invasion in duodenal or gastric metastasis [7], very few cases have found metastasis to the sigmoid or transverse colon by this mechanism [10]. It has been suggested that spread to the lymph nodes and lungs involves a one-step process compared to the pancreas, liver, or intestines which may involve a multistep process [4]. Thus, it is plausible that the patient’s RCC metastasized to the lung or liver first before hematogenous seeding to the colon and rectum. The patient’s larger cysts found prior to bilateral nephrectomy were on his right kidney, suggesting that hepatic involvement may have come first via direct invasion; however, because these cysts were benign, this is unlikely. Additionally, the patient’s shortness of breath preceded his change in bowel habits and rectal bleeding. Although the patient had routine imaging of his abdomen for underlying comorbidities, he did have not have imaging of his chest, which may have revealed an earlier diagnosis of pulmonary metastasis.

It is curious as to how one developed metastatic RCC approximately six years after bilateral nephrectomy in the setting of benign disease. Metastatic RCC to the gastrointestinal tract can present 7-19 years after nephrectomy [9]. Other reports have noted colonic metastasis in those with primary RCC, on average, 9.4 years after nephrectomy [9]. Individuals requiring transplantation and dialysis are at a high risk of developing RCC, regardless of the presence of ADPKD [5,12]. Although our patient was never treated with dialysis, he was on chronic immunosuppressive agents, which can increase the risk of malignancy post-transplantation [12]. In fact, a previous study demonstrated that the incidence of cancer is 10 times higher in renal transplant recipients than in dialysis patients due to immunosuppressant therapy [12]. Despite 15 years of immunosuppressant therapy, our patient never demonstrated signs of malignancy in his transplanted or native kidneys.

## Conclusions

Because the pathology reports confirmed benign cystic disease, there was no suspicion for malignancy for the six years following bilateral nephrectomy. Despite a close follow-up, the patient’s back pain and symptomatic anemia were attributed to his underlying osteoarthritis and ESRD, respectively, rather than evidence of metastatic disease. Additionally, the changes in his bowel habits were considered medication side effects and further delayed diagnosis. Although the mechanism of metastasis is truly unknown, we can postulate that it spread hematogenously or via direct invasion to either the lungs or liver before spreading to the colon and even in the absence of an apparent primary tumor. This is the first case in which a patient with benign cystic renal disease developed CCRC presenting as metastatic disease of the colon, rectum, liver, and lung.
